# Maternal Uncontrolled Anxiety Disorders Are Associated With the Increased Risk of Hypertensive Disorders in Japanese Pregnant Women

**DOI:** 10.14740/jocmr2284w

**Published:** 2015-08-23

**Authors:** Shunji Suzuki, Hiroki Shinmura, Masahiko Kato

**Affiliations:** a Department of Obstetrics and Gynecology, Japanese Red Cross Katsushika Maternity Hospital, Tokyo, Japan

**Keywords:** Pregnancy, Hypertensive disorders, Anxiety disorders, Depressive disorders, Self-interruption of medications

## Abstract

**Background:**

We examined the risk of hypertensive disorders in relation to maternal depressive and anxiety disorders which were diagnosed before or during early pregnancy in Japanese women.

**Methods:**

We reviewed the obstetric records of all Japanese singleton deliveries at ≥ 22 weeks’ gestation managed at the Japanese Red Cross Katsushika Maternity Hospital between 2009 and 2014. Potential risk factors for hypertensive disorders with maternal depressive and anxiety disorders were selected as follows: maternal age, parity, medications, self-interruption of medications and economic problems.

**Results:**

The incidence of hypertensive disorders did not increase in the pregnant women with depressive disorders compared with that in the normal control pregnant women (P = 0.96). However, the incidence of hypertensive disorders in the women with anxiety disorders was higher than that in the control women (odds ratio (OR): 2.61, 95% confidence interval (CI): 1.4 - 5.0, P < 0.01). In the women with anxiety disorders, 19% performed self-interruption of medications during pregnancy, and it was associated with the increased risk of hypertensive disorders (vs. no medication group, OR: 7.50, 95% CI: 1.5 - 38, P = 0.03; vs. medication group, OR: 16.0, 95% CI: 2.4 - 110, P < 0.01).

**Conclusions:**

Maternal uncontrolled anxiety disorders due to self-interruption of medications seemed to be associated with the increased risk of hypertensive disorders in Japanese pregnant women.

## Introduction

Mood and anxiety disorders are common, debilitating psychiatric illnesses that disproportionally affect women of childbearing age. Studies of the non-pregnant populations have shown that these mental disorders are associated with the development of hypertension [1-3]. In particular, individuals with depressive and anxiety disorders may be more prone to hypertension because of a presumed unhealthy lifestyle and associated changes in the autonomic nervous system, the hypothalamic-pituitary adrenal axis, and the immune system [3-5].

Depression and anxiety have been known to be the most common mental health problems in pregnancy [6, 7]. These have been reported to affect about 10 - 15 out of every 100 pregnant women [6]. Although the blood pressure regulation is substantially changed during pregnancy [8, 9], some observations have shown the similar associations in pregnant women indicating that these maternal mental disorders are associated with increased risk of preeclampsia [10, 11]. Few studies have evaluated that maternal depressive and anxiety disorders are risk factors for hypertensive disorders during pregnancy [10, 11]. For example, Qiu et al [10] reported that women with depressive or anxiety disorder prior to pregnancy have a 1.7 higher risk of the hypertensive disorder preeclampsia. In addition, Kurki et al [11] observed a significantly increased chance for preeclampsia if women were affected by depression, anxiety, or both during early pregnancy.

Based on these backgrounds, we examined the risk of hypertensive disorders in relation to maternal depressive and anxiety disorders which were diagnosed before or during early pregnancy in Japanese women.

## Methods

We reviewed the obstetric records of all Japanese singleton deliveries at ≥ 22 weeks’ gestation managed at the Japanese Red Cross Katsushika Maternity Hospital between 2009 and 2014. This retrospective study was approved by the Ethics Committee of Japanese Red Cross Katsushika Maternity Hospital. Demographic information and the characteristics of mental disorders were extracted from Japanese patient charts. Patients with chronic hypertension, renal disease, and systemic illnesses were excluded. Hypertensive disorders were defined as pregnancy-induced hypertension which was defined as blood pressure ≥ 140/90 mm Hg measured on two or more occasions at least 6 h apart with the patient at rest. Maternal depressive and anxiety disorders were diagnosed before or during early pregnancy by Japanese psychiatric specialists. Woman with economic problems was defined if she had received financial support for her delivery from the hospitalization assistance policy system by the Japanese Child Welfare Government as previously reported [12].

In this study, potential risk factors for hypertensive disorders with maternal depressive and anxiety disorders were selected as follows: maternal age, parity, medications, self-interruption of medications and economic problems.

Data are presented as n (%). Cases and controls were compared by χ^2^ or Fisher’s exact test for categorical variables. Differences with P < 0.05 were considered significant. Odds ratios (ORs) and 95% confidence intervals (CIs) were also calculated. Variables used in the multivariate model were those that on univariate analysis had shown significance (P < 0.05) toward association with increased risk of hypertensive disorders.

## Results

Between 2009 and 2014, for example, 9,461 Japanese pregnant women gave birth at our hospital. Of these, 95 (1.0%), 78 (0.8%) and 39 (0.4%) were diagnosed as having maternal depressive, anxiety and other mental disorders. Therefore, the rest 9,249 women (97.8%) were defined as control.

Table 1 shows the maternal age, parity and incidence of hypertensive disorders in the Japanese pregnant women with depressive and anxiety disorders. The incidence of hypertensive disorders did not increase in the women with depressive disorders (P = 0.96). However, the incidence of hypertensive disorders in the women with anxiety disorders was higher than that in the control group (OR: 2.61, 95% CI: 1.4 - 5.0, P < 0.01).

**Table 1:** Maternal Age, Parity and Incidence of Hypertensive Disorders in the Japanese Pregnant Women With Depressive and Anxiety Disorders

	Control	Depressive disorders	P-value	Anxiety disorders	P-value
Total	9,249	95		78	
Maternal age ≥ 35 years	3,470 (38%)	36 (38%)	0.98	24 (31%)	0.22
Nulliparity	5,734 (62%)	57 (60%)	0.69	48 (62%)	0.97
Hypertensive disorders	547 (6%)	6 (6%)	0.96	11 (14%)	< 0.01

Data are presented as number (%). P-values vs. control group.

Table 2 shows the incidence of hypertensive disorders in the Japanese pregnant women with depressive and anxiety disorders associated with the potential risk factors for hypertensive disorders. In the women with anxiety disorders, 10 of 52 women (19%) performed self-interruption of medications during pregnancy, and the self-interruption of medications was associated with the increased risk of hypertensive disorders (vs. no medication group, OR: 7.50, 95% CI: 1.5 - 38, P = 0.03; vs. medication group, OR: 16.0, 95% CI: 2.4 - 110, P < 0.01). The incidence of hypertensive disorders in the women with anxiety disorders without self-interruption of medications was 6.5% (6/93), which was not different from that in the control group (P = 0.83). In the women with depressive disorders, two of 70 women (3%) performed self-interruption of medications during pregnancy, and the self-interruption of medications was not associated with the increased risk of hypertensive disorders.

**Table 2:** Incidence of Hypertensive Disorders in the Japanese Pregnant Women With Depressive and Anxiety Disorders Associated With the Potential Risk Factors for Hypertensive Disorders

	Depressive disorders			Anxiety disorders		
	Hypertension (-)	Hypertension (+)	P-value	Hypertension (-)	Hypertension (+)	P-value
Total	89	6		67	11	
Maternal age						
< 35 years	32	4	0.29	49	6	0.14
≥ 35 years	57	2	Reference	18	5	Reference
Parity						
Nulliparity	53	4	Reference	41	7	Reference
Multiparity	40	2	0.97	26	4	0.85
Medication						
No	23	2	Reference	30	4	Reference
Yes	64	4	0.91	32	2	0.67
Self-interruption	2	0	0.32 and 0.23*	5	5	0.03 and < 0.01*
Economic problems						
No	85	6	Reference	56	10	Reference
Yes	4	0	0.6	11	1	0.87

Data are presented as number. *Versus medication group.

## Discussion

To date, several biological mechanisms may plausibly account for observed positive associations of maternal depressive and anxiety disorders and hypertensive risk. Increase in hypothalamic-pituitary-adrenal activity, a robust pathophysiological finding associated with depressive and anxiety disorders, is regarded as one important mechanism for observed associations between maternal psychiatric illness and adverse pregnancy outcomes [3-5, 10, 13, 14]. Altered plasma cortisol, β-endorphin corticotrophin releasing hormone, and serotonin concentrations in pregnant women with depressive and/or anxiety disorders have been also proposed [15, 16]. In addition, chronic systemic inflammation and related endothelial dysfunction associated with the pathogenesis of preeclampsia have been observed among individuals with clinical depression and depressive symptoms [17, 18].

In the current study, however, anxiety disorders were associated to an increased risk of hypertensive disorders in cases with self-interruption of medications only. In addition, depressive disorders were not associated with the incidence of hypertensive disorders during pregnancy.

In this study, 19% of the women with anxiety disorders performed self-interruption of medications during pregnancy. Women with mental disorders sometimes perform self-interruption of medications during pregnancy, and then the self-interruption of medications sometimes deteriorates their state of mental disorders [12]. Recently, it is possible to obtain the information about the impact of medications in perinatal outcomes in detail via the Internet and/or magazines. The users may misunderstand that the medications lead to the adverse outcomes of pregnancy due to their inappropriate information analyses. In particular, in the current study the pregnant women with anxiety disorders might have excessive anxiety for their medications during pregnancy, and the self-interruption of medications led to uncontrolled anxiety and adverse pregnancy outcomes such as the pathogenesis of hypertensive disorders through some possible mechanisms such as accelerated hypothalamic-pituitary-adrenal activity [3-5, 10, 13-17]. In the current study, on the other hand, the low rate of self-interruption of medications (3%) in the women with depressive disorders may be one of the major possibilities leading to the non-increased incidence of hypertensive disorders.

A number of important limitations should be considered when interpreting results from our study. First, the number of patients in this study may have been too small to obtain any significant result. In this study, there were only 10 women with self-interruption of medications for anxiety disorders. It is estimated that a definitive trial powered to detect the same increased risk of hypertensive disorders would require approximately 30 - 44 patients equally divided in the two groups with and without self-interruption of medications (two-tailed, α = 0.05, power = 80%). Second, we did not have information concerning the severity of psychiatric disorders and medication dosage especially in cases of depressive disorders. In addition, although we had detailed information about medication use, we cannot be certain that women receiving prescriptions during pregnancy actually took the medications [3, 10]. Therefore, a further prospective study may be needed to clarify the impact of self-interruption of medications on pregnancy outcomes in women with mental disorders.

### Conclusions

Maternal uncontrolled anxiety disorders due to self-interruption of medications seemed to be associated with the increased risk of hypertensive disorders in Japanese pregnant women.
